# Discordant antibody and cellular responses to *Pneumocystis* major surface glycoprotein variants in mice

**DOI:** 10.1186/1471-2172-13-39

**Published:** 2012-07-12

**Authors:** Lisa R Bishop, Daniel Helman, Joseph A Kovacs

**Affiliations:** 1 Critical Care Medicine Department, NIH Clinical Center, National Institutes of Health, 10 Center Drive, Bethesda, MD, 20892-1662, USA

**Keywords:** Antigenic variation, Immune response, Major surface glycoprotein, *Pneumocystis*

## Abstract

**Background:**

The major surface glycoprotein (Msg) of *Pneumocystis* is encoded by approximately 50 to 80 unique but related genes. Msg diversity may represent a mechanism for immune escape from host T cell responses. We examined splenic T cell proliferative and cytokine as well as serum antibody responses to recombinant and native *Pneumocystis* antigens in immunized or *Pneumocystis*-infected mice. In addition, immune responses were examined in 5 healthy humans.

**Results:**

Proliferative responses to each of two recombinant Msg variant proteins were seen in mice immunized with either recombinant protein, but no proliferation to these antigens was seen in mice immunized with crude *Pneumocystis* antigens or in mice that had cleared infection, although the latter animals demonstrated proliferative responses to crude *Pneumocystis* antigens and native Msg. IL-17 and MCP-3 were produced in previously infected animals in response to the same antigens, but not to recombinant antigens. Antibody responses to the recombinant *P. murina* Msg variant proteins were seen in all groups of animals, demonstrating that all groups were exposed to and mounted immune responses to Msg. No human PBMC samples proliferated following stimulation with *P. jirovecii* Msg, while antibody responses were detected in sera from 4 of 5 samples.

**Conclusions:**

Cross-reactive antibody responses to Msg variants are common, while cross-reactive T cell responses are uncommon; these results support the hypothesis that *Pneumocystis* utilizes switching of Msg variant expression to avoid host T cell responses.

## Background

*Pneumocystis* is a fungus that can cause severe pneumonia in immunosuppressed hosts, especially those with HIV infection, but that can also cause pulmonary infection that is cleared by a robust immune response in healthy hosts. While antibody responses are an important component of these responses, cell-mediated responses appear critical to the successful control of *Pneumocystis* infection [1,2]. *Pneumocystis* can infect a wide range of host species, and each host is infected by a genetically unique *Pneumocystis* species. Serologic studies among humans suggest widespread exposure to *Pneumocystis* at an early age [3].

**Figure 1:**
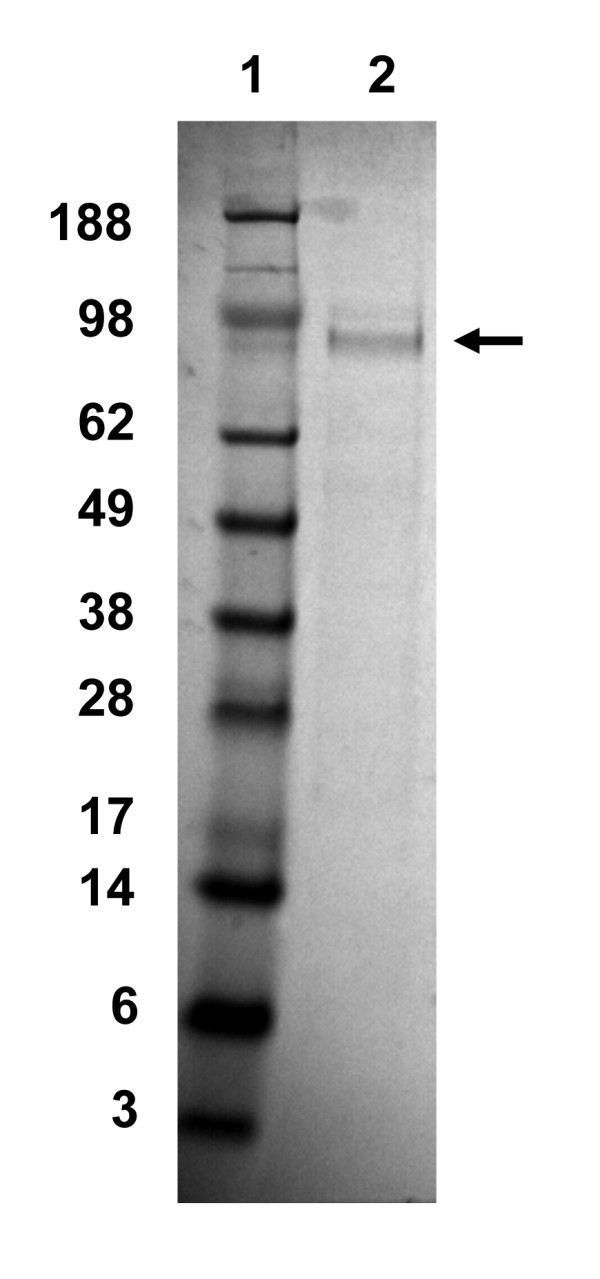
**SDS-PAGE analysis of native Msg.** Msg was purified as described in the methods and then analyzed by SDS-PAGE; the gel was stained with Coomassie Blue. Lane 2 shows Msg (arrow) with an approximate size of ~95 kilodaltons. Molecular weight markers are in lane 1, with their size in kilodaltons indicated on the left.

The most abundant surface protein is an ~95,000-110,000 MW protein known as the major surface glycoprotein (Msg, also called gpA; Figure 1) [4]. Msg appears to play a role in mediating adhesion to host cells, possibly through binding to fibronectin or vitronectin [5,6]. Msg is encoded by a family of approximately 50 to 80 genes which are related but distinct; each *Pneumocystis* species examined to date encodes a similar multi-copy gene family [7,8]. While multiple different Msgs may be expressed in a heavily infected immunosuppressed animal [9], a single organism can express only a single Msg [10-13]. *Pneumocystis* has developed a mechanism whereby there is a single expression site for *msg* genes [10-13]; the gene to be expressed must be placed downstream of and in-frame with the upstream conserved sequence (UCS), which serves as a leader for Msg and encodes a signal peptide for trafficking to the endoplasmic reticulum. The mechanism for switching of expressed genes is unknown but may involve gene conversion.

While Msg may play a functional role in *Pneumocystis* biology such as in facilitating adhesion, the presence of multiple Msg variants strongly suggests that *Pneumocystis* has developed a mechanism for antigenic variation, similar to other organisms such as trypanosomes or *Borrelia*[8,14]. Antigenic variation could potentially allow persistence of infection or facilitate re-infection, contributing to the ubiquitous nature of this organism. Although *Pneumocystis* is a pathogen of immunosuppressed hosts, *Pneumocystis* can infect and induce a brisk and effective immune response in immunocompetent hosts [15,16]. Antigenic variation presumably evolved to evade host responses in the latter, given that immunosuppressed hosts would likely rarely be encountered in nature.

Antigenic variation in many pathogens is designed to avoid humoral immune responses [14]. In *Pneumocystis*, although antibodies play a role in protection, cellular immune responses, especially CD4+ T cell responses, are critical for clearing infection and providing protective immunity [1,2]. We hypothesized that a primary benefit of maintaining a broad Msg repertoire is to allow switching of the expressed Msg variant to evade cellular immune responses, especially CD4+ T cell responses, given that *Pneumocystis* is exclusively an extracellular pathogen. The current study was undertaken to address this hypothesis by examining: 1. cellular and humoral responses in mice to two recombinant Msg variants following immunization with recombinant proteins or native *Pneumocystis* antigens, as well as following natural infection; and 2. cellular proliferative and humoral responses in humans to a recombinant Msg variant.

## Results

In order to study immune responses in mice to individual Msgs, we expressed 2 recombinant Msg variants, 107 and 119, which differed from each other by 11%, in *E. coli*. Because of difficulties in expressing the entire Msg as a single protein, we expressed each variant in two overlapping fragments, corresponding approximately to the amino and carboxyl halves of the full-length protein. In addition, we expressed each fragment in multiple vectors, one of which was used for immunization studies, and another for in vitro assays. This eliminated cross-reactive immune responses to the vector-encoded portion of the recombinant protein which we identified in preliminary studies.

We utilized proliferation to examine cellular T cell responses to *Pneumocystis* antigens; this will primarily represent CD4+ T cell responses to protein (vs. peptide) antigens. In a subset of animals we also examined culture supernatant cytokine levels following antigenic stimulation. Attempts to detect intracellular cytokine responses, including interferon-gamma, interleukin-4, interleukin-17, and tumor necrosis factor-alpha, by flow cytometry were unsuccessful despite numerous attempts using a variety of *Pneumocystis* antigens.

**Figure 2:**
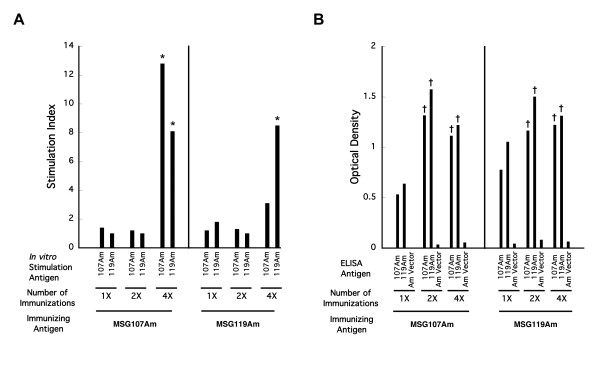
**A. Cell proliferation following immunization with recombinant*P. murina*Msg proteins MSG107Am and MSG119Am.** Splenocytes from animals that were immunized with these recombinant Msg proteins were cultured in triplicate with each of the recombinant Msg proteins and (as a control) with recombinant protein encoded by the empty vector (without Msg). Groups of 4 animals received one, two or four injections with each antigen, as indicated by 1X, 2X, and 4X. Bars represent the stimulation index (SI) (geometric mean of results for Msg-stimulated response divided by empty vector-stimulated response). **B.** Antibody reactivity to the Msg variants measured by ELISA. Serum samples were run in duplicate for each antigen. Bars represent the mean optical density for 4 animals per immunization group. Unpaired t tests were used to compare the results for immunized animals to control (unimmunized or adjuvant alone) animals. Statistical significance is indicated by the following symbols: *, p ≤ 0.05; ‡, p ≤ 0.01; †, p ≤ 0.001.

We initially immunized separate sets of 4 mice 1, 2, or 4 times with the amino fragment of each of the two recombinant Msg variants 107 and 119 (20 μg/immunization), because there was less conservation in the amino (86%) compared to the carboxyl (91%) fragments. We examined proliferative and antibody responses to both variants; results are shown in Figure 2. Splenocytes from one of 4 mice immunized with a single dose of Msg119Am proliferated in response only to the immunizing antigen. No proliferation was seen to either the immunizing or the non-immunizing variant fragment following 2 immunizations with either amino fragment. Following the 4^th^ immunization, proliferative responses to the immunizing antigen were seen in splenocytes from 6 of 8 animals, while proliferation to the non-immunizing Msg amino fragment was seen in splenocytes from 5 of these 8 animals. In contrast, antibody responses as measured by ELISA were seen in 5 of the 8 animals to both fragments following the first immunization, and in 16 of 16 animals to both fragments following 2 or 4 immunizations; optical densities were similar to both fragments regardless of whether 107 or 119 was used for immunization*.* Antibody responses to crude *P. murina* antigens were seen only in animals with antibody responses to the Msg variants (20 of 24 animals). Thus, cross-reactive proliferative responses were seen only following multiple immunizations, while cross-reactive antibody responses were seen after a single immunization. No immune responses were seen to the recombinant Msg fragments or crude *P. murina* antigens in control animals immunized with adjuvant alone (data not shown).

**Figure 3:**
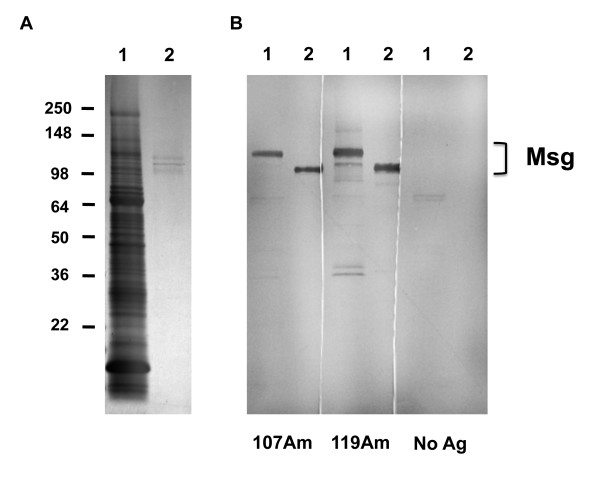
**Western blot analysis of antibody responses generated following immunization with recombinant Msg.** Panel **A** shows a Coomassie blue-stained gel and panel **B** shows western blots using sera from 2 immunized mice and 1 control mouse. For both the gel and the blots, lane 1 utilized a crude *Pneumocystis* antigen extract, while lane 2 utilized purified Msg. For the western blots, the serum used to probe the blots is shown under the blot. 107Am is from an animal that received 4 immunizations with MSG107Am, and 119Am is from an animal that received 4 immunizations with MSG119Am. No Ag is from an animal immunized with adjuvant alone. Brackets indicate the location of Msg. The Msg in purified preparations is smaller than in crude preparations because lyticase treatment, which is used to solubilize Msg, results in a decrease in the apparent molecular weight of Msg.

To verify that the antibody responses to recombinant proteins recognized native Msg, we utilized western blots with either crude *Pneumocystis* antigens or purified native Msg as the antigen. Using sera from animals immunized with the amino recombinant fragments, we were able to demonstrate reactivity with Msg in both antigen preparations in 2 of 2 animals tested. Figure 3 shows results using sera from animals immunized with 107Am or 119Am.

**Figure 4:**
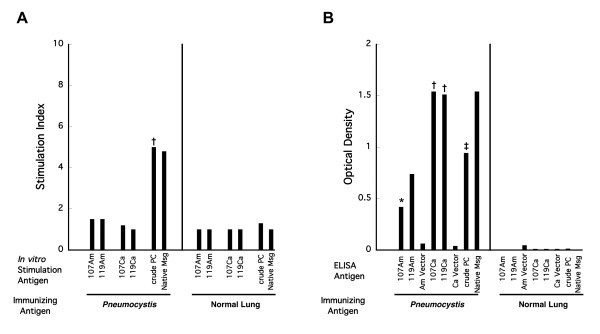
**A. Cell proliferation following immunization with crude*Pneumocystis*antigens.** Splenocytes from animals that were immunized four to five times with crude antigens were cultured in triplicate with recombinant *P. murina* Msg proteins (n = 5 except n = 3 for MSG119Am), crude *Pneumocystis* antigens (n = 5), and purified native Msg (n = 2). Non-immune animals or animals immunized with normal lung antigen were used as controls and splenocytes were cultured with the same antigens (n = 4 for all except n = 3 for MSG119Am and n = 1 for purified native Msg). Bars represent the stimulation index (SI) as compared to appropriate vector without Msg for recombinant antigens, normal lung antigens for crude *Pneumocystis* antigens and no antigen for native Msg. **B**. Antibody reactivity to each of the antigens measured by ELISA. Serum samples were run in duplicate for each antigen. Each bar in B represents the mean optical density for each antigen in *Pneumocystis* antigen immunized or control immunized animals (the same number of animals were used as for A). Unpaired t tests were used to compare the results for immunized animals to control (normal lung immunized) animals. Statistical significance is indicated by the following symbols: *, p ≤ 0.05; ‡, p ≤ 0.01; †, p ≤ 0.001. For native Msg statistical significance was not calculated due to the small number of animals.

We next examined proliferative and antibody responses following immunization with crude *P. murina* antigens. Msg is the most abundant protein in *Pneumocystis* extracts, and in immunosuppressed animals, which are the source of this *Pneumocystis*, multiple Msg variants are expressed [9]. Splenocytes from immunized animals (n = 5) did not proliferate in response to challenge with either the amino or the carboxyl fragment of either MSG107 or MSG119, but did proliferate (5 of 5 animals) to crude *P. murina* antigens, which were used to immunize the animals (Figure 4). Proliferative responses were also seen to purified native Msg in splenocytes from immunized animals (2 of 2) but not in a control animal. In contrast to this, antibody responses were seen to both amino (4 of 5 for 107Am and 3 of 3 for 119Am) and carboxyl fragments of MSG107 and MSG119, as well as to crude *P. murina* antigens, in all 5 animals. Antibody responses were also seen to purified native Msg (2 of 2). It is noteworthy that the antibody responses were greater (higher OD) to both carboxyl fragments, which are more highly conserved, than to the amino fragments. The antibody responses demonstrate that the animals were exposed to, and were able to mount an immune response to, Msg in the crude antigen preparations.

**Figure 5:**
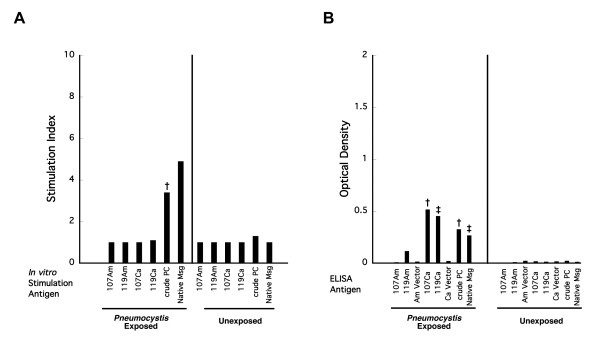
**A. Cell proliferation following*Pneumocystis*infection.** Blood and spleen cells were obtained 10 to 12 weeks after initial exposure to *Pneumocystis*-infected seeders, a time by which *Pneumocystis* infection has typically developed and been cleared in healthy, immunocompetent animals. Splenocytes from these animals were cultured with recombinant *P. murina* Msg proteins (n = 9 except n = 7 for MSG119Am), crude *Pneumocystis* antigens (n = 9), and purified native Msg (n = 2). Splenocytes from unexposed control animals were cultured with the same antigens (n = 4 for all except n = 3 for MSG119Am and n = 1 for native Msg). Bars represent the stimulation index (SI) as compared to proliferation to appropriate vector without Msg for recombinant antigens and normal lung antigens for crude *Pneumocystis* antigens and no antigen for native Msg. **B**. Antibody reactivity to each of the antigens measured by ELISA. Each bar represents the mean optical density of the *Pneumocystis* exposed or control animals (the same number of animals were used as for A). Of note, 1 of 7 *Pneumocystis* exposed animals had antibodies to MSG119Am. Unpaired t tests were used to compare the results for exposed animals to control (unexposed) animals. Statistical significance is indicated by the following symbols: *, p ≤ 0.05; ‡, p ≤ 0.01; †, p ≤ 0.001. For native Msg (left panel) statistical significance was not calculated due to the small number of animals.

Because immunization with either recombinant or crude antigen preparations presents an artificial encounter with *Pneumocystis* antigens, we wanted to examine immune responses following natural infection. Since immunosuppressed animals have inadequate immune responses to *Pneumocystis*, which allow the organism to replicate in an uncontrolled or poorly controlled manner, we utilized a model in which healthy animals develop *Pneumocystis* infection following exposure to an already infected seeder animal. In this model, infection peaks at ~5-6 weeks following exposure, and is typically cleared by 10 weeks [15]. Splenocytes from exposed mice proliferated in response to crude *Pneumocystis* antigens in 7 of 9 animals and to native Msg in 2 of 2 animals, but none of the animals showed proliferation to any of the 4 recombinant Msg protein fragments (Figure 5). Antibody responses were again seen to the carboxyl recombinant Msg fragments (9 of 9 and 8 of 9 for MSG107 and MSG119, respectively) as well as to crude *Pneumocystis* antigen and native Msg (8 of 9 for each), but not to the amino fragments (0 of 9 and 1 of 7 for MSG107 and MSG119, respectively), again indicating that these naturally infected animals were exposed to Msg.

**Figure 6:**
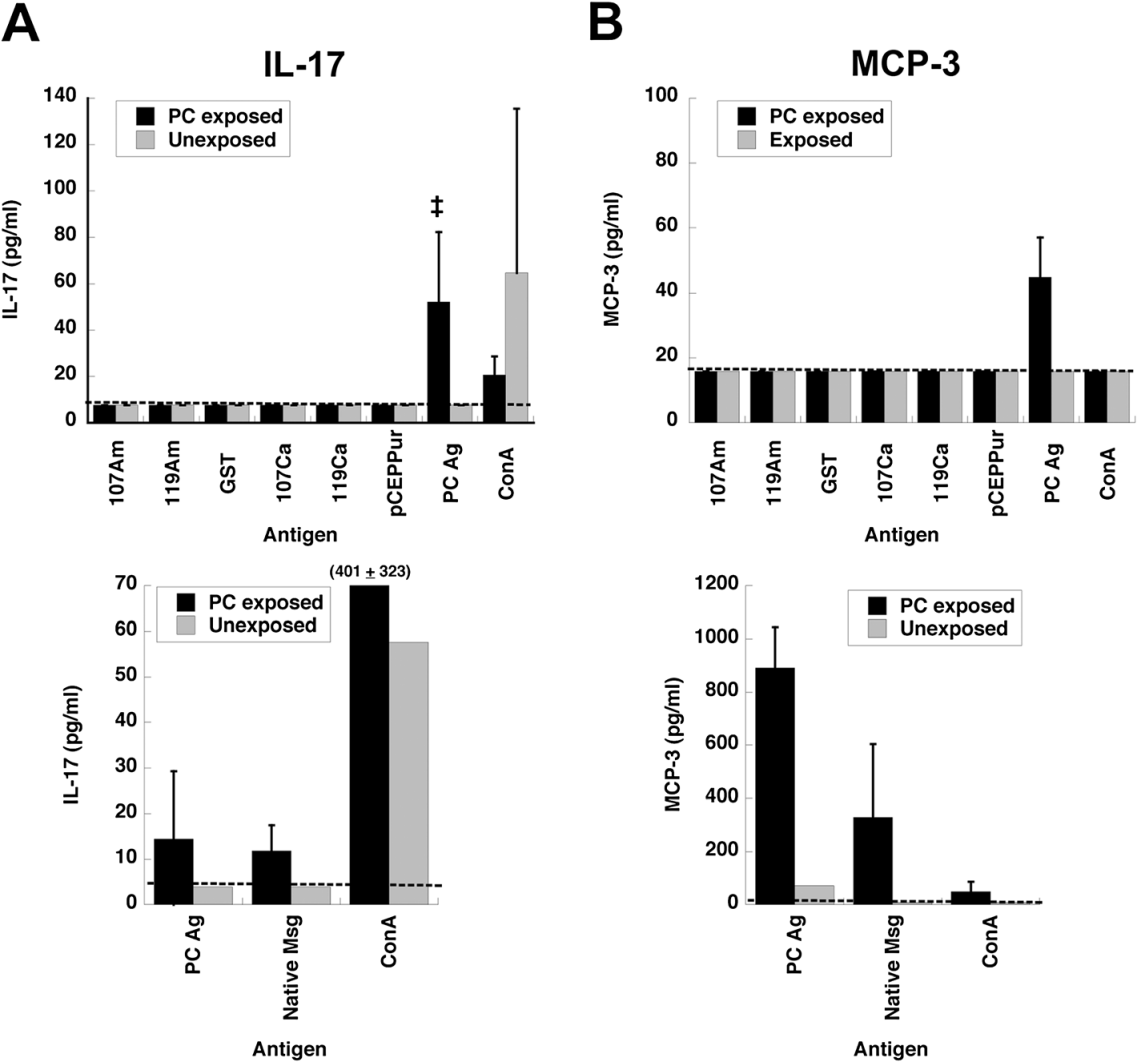
**Analysis of cytokines secreted by splenocytes from*Pneumocystis*-infected animals.** Splenocytes were obtained 10 to 12 weeks after exposure, by which time *Pneumocystis* infection had typically cleared, and were cultured with recombinant Msg proteins, crude *Pneumocystis* antigens, or native Msg. **A** shows the results for IL-17, and **B**, the results for MCP3. Results for IL-17, top, represent the average of 7 *P. murina* exposed animals or 3 unexposed control animals (except for concanavalin A, n = 2) and bottom, the average of 2 *P. murina* exposed animals or results for 1 unexposed control animal. Results for MCP-3, top, represent the average of 4 *P. murina* exposed animals (except n = 3 for crude *P. murina* antigen) or 2 unexposed control animals and bottom, the average of 2 *P. murina* exposed animals (except concanavalin A, n = 1) or results for 1 unexposed control animal. The dashed line in each panel indicates the lower limit of detection; the error bars indicate the standard deviation. Unpaired t tests were used to compare the results for exposed animals to control (unexposed) animals. Statistical significance is indicated by the following symbols: *, p ≤ 0.05; ‡, p ≤ 0.01; †, p ≤ 0.001. Statistical significance was not calculated for the bottom panels due to the small number of animals. Although the results for MCP-3 (top panel) did not reach statistical significance (p = 0.054), all 3 animals had elevated MCP-3 levels in response to crude *Pneumocystis* antigen, and both animals in the bottom panel showed elevated levels to both crude *Pneumocystis* antigen and native Msg. In the top panels, error bars are not shown for the results that are below the lower limit of detection, since results for all animals in each group were below that value. In the bottom panels, error bars are not shown for the single unexposed animal.

As an additional marker of CD4 responses, we examined in vitro cytokine production. Among the cytokines examined, IL-17 and MCP-3 were consistently secreted by splenocytes from animals infected with *P. murina* when cultured with *P. murina* Ag or native Msg antigen, but not with the recombinant Msg variants (Figure 6). These responses represented memory responses, as no cytokine production was seen in cells from uninfected control animals cultured with the same antigens. Among the other cytokines, results with crude antigen or native Msg were variably positive (data not shown) but in no case were they produced in response to the recombinant antigens.

Serologic studies have found that most humans have been exposed to *Pneumocystis* at a young age and have detectable antibodies to Msg. To see if PBMCs from healthy humans can proliferate to recombinant Msg, cells from 5 volunteers were cultured with HuMSG14Am, HuMSG14Ca, concanavalin A or tetanus toxoid. No patient demonstrated proliferation to either HuMSG fragment after 5 days, while all samples proliferated in response to concanavalin A or tetanus toxoid. Moreover, sera from 4 of the 5 volunteers were positive in an ELISA utilizing HuMSG14Ca, while the 5^th^ volunteer had high non-specific reactivity.

## Discussion

This study has demonstrated that while cross-reactive proliferative responses to two distinct recombinant Msg variants were induced by multiple immunizations with recombinant Msg protein, proliferative responses to these recombinant proteins were not seen following immunization with crude *Pneumocystis* antigens, which contain Msg, or following natural infection in a healthy host. Further more, these proteins did not induce IL-17 and MCP-3 production in infected animals, unlike the crude antigen or purified native Msg. In contrast, nearly all animals developed antibody responses to the recombinant Msg fragments, indicating that they recognized conserved B cell epitopes in Msg. Since the cell-mediated (proliferation and cytokine) responses to extracellular proteins represent primarily CD4+ T cell responses, these data are thus consistent with the hypothesis that Msg variants exhibit antigenic variation that evades CD4-mediated T cell responses rather than B cell responses. Because in vitro culture and genetic manipulation of *Pneumocystis* is not currently possible, more direct methods for addressing this question, such as utilization of organisms that express a single *msg* gene, are currently not feasible, and thus indirect methods, as in the present study, must be utilized.

CD4 cells are critical to controlling infection with *Pneumocystis*, and CD4 responses have previously been shown to target Msg. Further, immunization with Msg has been shown to induce proliferative responses [17] but such immunization did not provide complete protection against *Pneumocystis* infection [17,18].

Western blot studies demonstrated that the antibodies generated following immunization with recombinant proteins recognized native Msg that was purified or present in crude antigen extracts. Thus conformational differences or differences in post-translational modifications that may exist between native and recombinant proteins did not prevent development of cross-reactive antibodies. While T cell responses were not detected, this cannot be due to conformational differences since both CD4 and CD8 cells recognize processed peptides of ~8-10 amino acids that are not dependent on conformation.

We initially examined immune responses following immunization with recombinant Msg variants, and found cross-reactivity in both proliferation and antibody responses after 4 immunizations, but only antibody responses following a lower exposure (1 or 2 immunizations). These initial studies also demonstrated that we could detect in vitro proliferation to the recombinant Msg proteins. Of note, the animal that showed proliferation after only 1 immunization proliferated only to the immunizing Msg variant, but developed antibodies that reacted with both Msg variants. No animals showed proliferative responses in the absence of antibody responses. These data suggest that CD4 responses, as measured by proliferation, require repeat or prolonged exposure to Msg. Such repeated exposure may allow for the expansion of a small number of initially responsive T cells or recruitment of new T cells recognizing additional Msg epitopes.

Interestingly, animals immunized with crude *P. murina* antigens demonstrated proliferative responses to the immunizing antigen and to purified native Msg, but not to either of the recombinant Msg variants, including the more conserved carboxyl portions (MSG107Ca and MSG119Ca). A likely explanation for this observation is that the genes encoding MSG107 and MSG119 were not expressed by the *P. murina* organisms used for preparation of the crude antigen, since only 1 of the 50 to 80 Msg genes is expressed by a *P. murina* organism at a time [10-13]. While some of these cellular responses may also be to other *P. murina* antigens, possibly Kexin or other unidentified *P. murina* antigens [19-23], proliferation following exposure to native Msg clearly demonstrates that cellular responses were directed in part against Msg. Although the number of animals studied using native Msg was small, precluding statistical analysis, the results were consistent with and supportive of those using crude antigen.

Finally, naturally infected animals showed production of IL-17 and MCP-3 and proliferation to crude *P. murina* antigen and to native Msg protein but again lacked cross-reactive proliferative responses to either the amino or carboxyl portions of Msg variants. Antibody responses were seen to the more conserved carboxyl fragment in most animals. Because we are not able to identify Msg variants that are expressed by *Pneumocystis* in individual naturally infected immunocompetent animals, we could not directly examine proliferative responses to the Msg variants that were expressed by the infecting organism, nor could we compare their sequences to those of the recombinant Msgs that were utilized in the in vitro assays.

Our results are in contrast to those of Theus et al. [24] who demonstrated proliferative responses to a recombinant Msg protein as well as to native Msg, using splenocytes and T cell clones from naturally infected rats. It is possible that the recombinant protein used in those studies was identical to or more closely related to the Msg variants expressed by *Pneumocystis* during infection of those animals. Interestingly, immunization with native or recombinant Msg in rats provided partial protection from *Pneumocystis* pneumonia, leading to a lower organism burden, while immunization with native Msg in mice was not protective, though antibody responses were induced in both studies [17,18].

We attempted to detect antigen-specific responses by intracellular cytokine staining using *Pneumocystis* antigens that were positive in the proliferation assays, but were unable to detect such responses despite utilization of a variety of assay conditions. We are aware of no publication to date that has successfully identified *Pneumocystis*-specific responses using this assay. This may be in part a function of the host-specific response to *Pneumocystis*, since such responses have been easily identified for many other pathogens [25].

Serologic studies have demonstrated that most humans are exposed to *Pneumocystis* at an early age [3]; consistent with this, most humans, including those included in the current study, have antibodies to Msg. While we were not able to perform the same spectrum of studies in humans as in mice, we were able to demonstrate, in previously infected humans (based on serology), absence of proliferative responses to a recombinant Msg to which they maintained antibody responses. Although the total number of patients studied is small, and while it’s possible that the proliferative T cell responses have waned over time, when combined with the animal data these results support the concept that antigenic variation helps avoid T cell, but not B cell, mediated immune responses. Very few studies have examined immune responses to *Pneumocystis*-specific antigens in humans, and all published studies have utilized rodent-derived antigens, which raises concerns that detected responses are non-specific, targeting xenogeneic, host-derived antigens, rather than being *Pneumocystis*-specific [26-28]. Additional studies such as ours, that utilize purified or recombinant *P. jirovecii-*derived antigens, are clearly needed to better understand the interaction between *Pneumocystis* and humans.

## Conclusions

This study has shown that in mice, cellular responses (proliferation and cytokine production) and antibody responses to variants of the Msg of *Pneumocystis* are discordant. Cross-reactive antibody responses occur following immunization with recombinant or native antigens, as well as following natural exposure to *Pneumocystis*, while cross-reactive cellular responses are not seen following immunization with native antigens or following natural exposure. The *Pneumocystis* genome contains multiple *msg* gene variants, only one of which is expressed in an organism, which thus confers upon *Pneumocystis* the potential for antigenic variation to avoid host immune responses. Given that cross-reactive cellular responses were not seen following immunization with native antigens or following natural exposure, while cross-reactive antibody responses were seen in these same animals (demonstrating response of their immune system to Msg), our results support the concept that antigenic variation in *Pneumocystis* has evolved to evade cellular rather than antibody responses. This is consistent with the observation that *Pneumocystis* pneumonia occurs almost exclusively in hosts with predominantly cellular immunodeficiencies, such as HIV infection, reflecting the importance of cellular immune mechanisms in control of this infection.

## Methods

### Animals

Healthy Balb/c, Balb/c *scid*, and C57 black (C57BL) mice were obtained from the National Cancer Institute, and CD40 ligand knock-out (CD40L-KO, strain B6, 129 S-Tnfsf5^tm1lmx^) mice were obtained from Jackson Laboratory. BALB/c *scid* and CD40L-KO mice were subsequently bred at the NIH. Mice were housed in microisolator cages and kept in ventilated racks. All animal work was performed under an NIH Clinical Center Animal Care and Use Committee-approved protocol.

### Human samples

Serum and peripheral blood mononuclear cells (PBMCs) were obtained from anonymous healthy NIH blood bank volunteers under IRB approved protocols. Guidelines of the National Institutes of Health and the US Department of Health and Human Services were followed in the conduct of these studies.

### *P. murina* antigen preparation

Crude antigens for immunization and cell proliferation assays were prepared from *scid* lungs heavily infected with *P. murina* (as determined by Diff Quik staining) or from uninfected wild-type lungs by homogenization in PBS (0.25 g/mL) using a Polytron (Omni International) followed by sonication. The homogenate was centrifuged for 10 minutes at 20,000 g, and the supernatant was collected. *Pneumocystis* antigens for ELISA were prepared in a similar manner from *Pneumocystis* organisms that were partially purified by Ficoll-Paque (GE Healthcare) gradient centrifugation. Glycerol was added to a final volume of 10% and the preparations were filter sterilized. Protein concentrations were determined using BCA (Pierce Biotechnologies).

Native Msg was prepared from partially purified *P. murina* organisms that were treated with lyticase (Sigma-Aldrich) overnight at 37°C [4]. Following centrifugation, Msg was purified from the supernatant using a concanavalin A-based glycoprotein isolation kit (Thermo Scientific). The buffers were changed to PBS plus 10% glycerol using a Microcon centrifugal filter device (Millipore). The protein concentration was determined by BCA or by measuring the absorbance at 280 nm using a NanoDrop spectrophotometer (Thermo Scientific) and purity was determined by SDS-PAGE (Figure 1).

### Recombinant Msg antigen preparation

DNA extracted from *P. murina*–infected *scid* mouse lung tissue was used as a template to amplify major surface glycoprotein (*msg*) genes of *P. murina* (which do not contain introns) by PCR. To facilitate expression, the region encoding a hydrophobic tail was excluded from the amplicon. PCR products were cloned into the pCR2.1 TA cloning vector (Invitrogen). *msg* clones were sequenced and two gene variants with among the highest differences (89% identity) at the amino acid level were chosen for protein expression: MSG107 (Accession # JF308632) and MSG119 (Accession # JF308633). Because of difficulty in expression of full-length Msg proteins, each *msg* was cloned into 2 fragments, MSG-Am and MSG-Ca, which encode the amino portions and carboxyl portions of the Msg proteins, respectively. MSG107Am and MSG119Am were 86% identical at the amino acid level and MSG107Ca and MSG119Ca were 91% identical at the amino acid level. Each of the MSG-Am gene fragments was cloned into bacterial expression vectors pET32a (Novagen), pGEX-6P-1 (GE Healthcare, Life Sciences) and pMALc2X (New England Biolabs), modified with the addition of a histidine tag and kindly provided by Dr. Peter Burbelo. MSG-Am proteins were expressed in BL21-CodonPlus (DE3)-RIL cells (Stratagene) and then purified using a Ni-NTA agarose (Qiagen) column or GST Purification module (GE Healthcare, Life Sciences) [29]. Because of difficulty in expression in bacterial systems, the MSG-Ca proteins were cloned into a modified pCEPPu/BM40 eukaryotic expression vector [30], kindly provided by Dr. Craig Rhodes, expressed in Cos1 cells and purified using Ni-NTA agarose (Qiagen). The buffers were changed to PBS, 10% glycerol and the proteins were concentrated using Amicon Ultra or Centricon centrifugal filter devices (Millipore). Endotoxin was removed from proteins expressed in *E. coli* systems using Triton X-114 [31]; endotoxin levels in the final preparations were below 0.05 EU/ml as measured by QCL-1000 Endpoint Chromogenic LAL Assay (Lonza). Recombinant Msg protein concentrations were determined using BCA.

HuMSG14 (GenBank accession no. AF033209) [32] a *P. jirovecii msg* gene, was cloned in 2 fragments, HuMSG14Am (encoding the amino portion) and Hu-MSG14Ca (encoding the carboxyl portion) into pET32a, and expressed in BL21-CodonPlus (DE3)-RIL cells (Stratagene). Recombinant Msg proteins were purified using a Ni-NTA agarose (Qiagen) column as previously described [33].

### Immunizations

Healthy Balb/c or C57BL mice were immunized subcutaneously with approximately 475 μg crude *P. murina* or normal lung antigen, or 20 μg recombinant Msg antigen using Freund’s complete adjuvant, and boosted with one to four additional injections using Freund’s incomplete adjuvant. Approximately 2 weeks following the last injection, mice were sacrificed and their spleens and blood were collected.

### *P. murina* exposure

To replicate natural infection, healthy C57BL mice were co-housed with a *P. murina*-infected (*scid* or CD40L-KO) seeder mouse or in control cages without a seeder mouse. After 10 to 12 weeks of exposure, by which time healthy, immunocompetent mice have typically cleared *Pneumocystis* infection and have detectable immune responses [15,16], mice were sacrificed and their spleens and blood were collected.

### Cell proliferation assay

Mouse spleen cells were cultured in 96 well plates (100,000 cells per well) with concanavalin A (2.5 μg/ml) for 4 days, and *P. murina* antigen (100 μg/ml), normal mouse lung antigen (100 μg/ml), recombinant Msg antigens (5 μg/ml), recombinant vector antigen (recombinant protein from the vector with no *msg* insert) (5 μg/ml), native msg antigen (2.5 μg/ml) or no antigen for 5 days. Similarly, human PBMCs were cultured in 96 well plates (100,000 cells per well) with concanavalin A (25 μg/ml) for 4 days or tetanus (1:10 dilution) for 5 days, and with recombinant HuMSG14Am or HuMSG14Ca (0.1 to 10 μg/ml) or no antigen for 5 days. To demonstrate that native or recombinant Msg proteins are not toxic to cells, in some experiments human PBMCs or mouse splenocytes were cultured with concanavalin A plus recombinant Msg antigens or native Msg. No inhibition of proliferation was seen in these studies.

Cell proliferation was measured after 4 to 5 days of culture using tritiated thymidine incorporation (triplicate wells) or CellTiter-Glo Luminescent Cell Viability Assay (duplicate or triplicate wells; Promega) [34]. For the former, 0.4 μCi tritiated thymidine was added to each well, cells were incubated at 37°C for an additional 4 hours, then harvested onto FilterMAT (Skatron Instruments) using a Tomtec Mach II M cell harvester, and counted using a scintillation counter (Beckman). CellTiter Glo was used according to the manufacturer’s instructions. Luminescence was read on a Centro LB 960 luminometer (Berthold Technologies). Results are presented as stimulation index, which represents the fold-change compared to the respective vector with no insert for recombinant antigens, normal lung antigen for crude *Pneumocystis* antigen, and no antigen for native msg; a stimulation index of 3 or greater was considered positive.

### Cytokine secretion

Fresh mouse spleen cells were cultured in 24 or 96 well plates at 0.5-1.0 X10^6^ cells per ml with recombinant Msg antigens, *P. murina* antigen, native Msg antigen, or concanavalin A at the same concentrations used in the cell proliferation assays. Cells were incubated at 37°C for 72 hours and the supernatants were harvested and stored at -80^o^C. Secreted cytokines were detected in the cell culture supernatants using Procarta Cytokine Assay Kits (Affymetrix) using a Bio-Plex 200 instrument (Bio-Rad). The following cytokines were assayed: IL-1 beta, IL-2, IL-6, IL-10, IL12p70, IL-17, IL-23, interferon-gamma, GM-CSF, TNF-alpha, MCP-3, and IP-10; IL-4, IL-15, IL-21, and KC were assayed in a subset of samples.

### Immunoblot

Approximately 34 μg crude *P. murina* antigen and 0.3 μg native msg antigen were run on a 4-20% Tris-Glycine SDS-PAGE gel (Invitrogen). Proteins were stained with Coomassie blue or were transferred onto a nitrocellulose membrane (Invitrogen). After blocking, the membranes were incubated with mouse sera diluted 1:100 followed by peroxidase conjugated anti-mouse IgG antibody (Jackson ImmunoResearch Laboratories). Reactivity was detected using 3,3’,5,5’-Tetramethylbenzidine (TMB) substrate (Sigma-Aldrich).

### ELISA

Anti-*P. murina* serum antibodies were measured by ELISA as previously described [15]. Briefly, native Msg (0.5 μg/ml), recombinant Msg (2.5 to 5 μg/ml), or *Pneumocystis* antigens prepared from partially purified *P. murina* organisms (1.6 μg/ml) were bound to duplicate wells in a 96 well plate at 37°C for 2 hours. After washing, the wells were blocked with 5% milk plus 5% goat serum in PBS at 37°C for 2 hours and then overnight at 4°C. The wells were then incubated with 100 μl mouse serum diluted 1:100 in 5% milk plus 5% goat serum in PBS with 0.05% Tween 20 for 1 hour at room temperature, and then after washing were incubated with HRP-conjugated goat anti-mouse IgG (heavy + light chains) (Jackson ImmunoLabs) diluted 1:1000 in 5% milk plus 5% goat serum in PBS with 0.05% Tween 20 for 1 hour at room temperature. After washing, 100 μl OPD substrate (Sigma) was added to each well. Optical densities (450 nm) were read at 1 hour. Anti-*P. jirovecii* Msg antibodies were measured by ELISA as previously reported [33].

### Statistical analysis

For analysis of the proliferation data, the results were log_2_ transformed to normalize the data, and a two-sample *t*-test assuming unequal variance was performed using Excel (Microsoft). The geometric mean data were back-transformed for presentation of the results. For analysis of the ELISA and cytokine results, the same *t*-test analysis was performed on untransformed data. Values below the detection of the cytokine assays were set at the detection limit for this analysis.

## Abbreviations

Msg, Major surface glycoprotein; PBMCs, Peripheral blood mononuclear cells.

## Competing interest

The authors have no competing interests.

## Authors’ contributions

LRB helped design the study, performed the experiments, and drafted the manuscript; DH participated in preparation of the recombinant antigens; JAK conceived of the study, and participated in its design and coordination and helped to draft the manuscript. All authors read and approved the final manuscript.
